# Supplementary figures and images for: Brief early-life motor training induces behavioral changes and alters neuromuscular development in mice
Source: PLoS Biol. 2025 Apr 21;23(4):e3003153. doi: 10.1371/journal.pbio.3003153 (PMC12052215; doi:10.1371/journal.pbio.3003153)

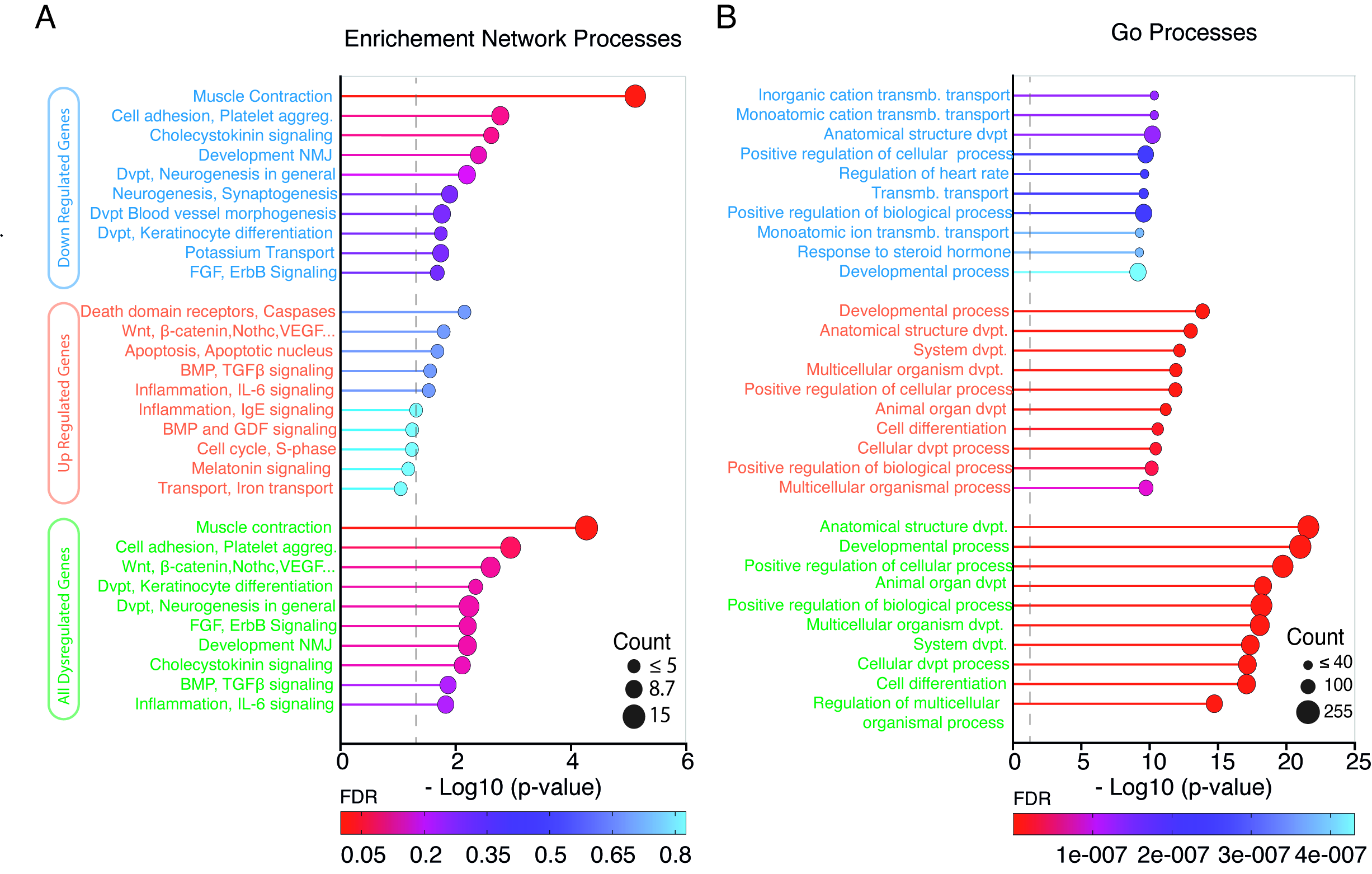

Supplement: S1 Fig — A. Top 10 GO enrichment network processes and B. Top 10 of GO processes, associated with upregulated genes, downregulated genes, or all the dysregulated genes in trained mice as ranked by the p-value (dashed line). The size of the circles reflects the number of genes involved in the processes described, while the color scale indicates the FDR value. (TIF) [file pbio.3003153.s001.tif]

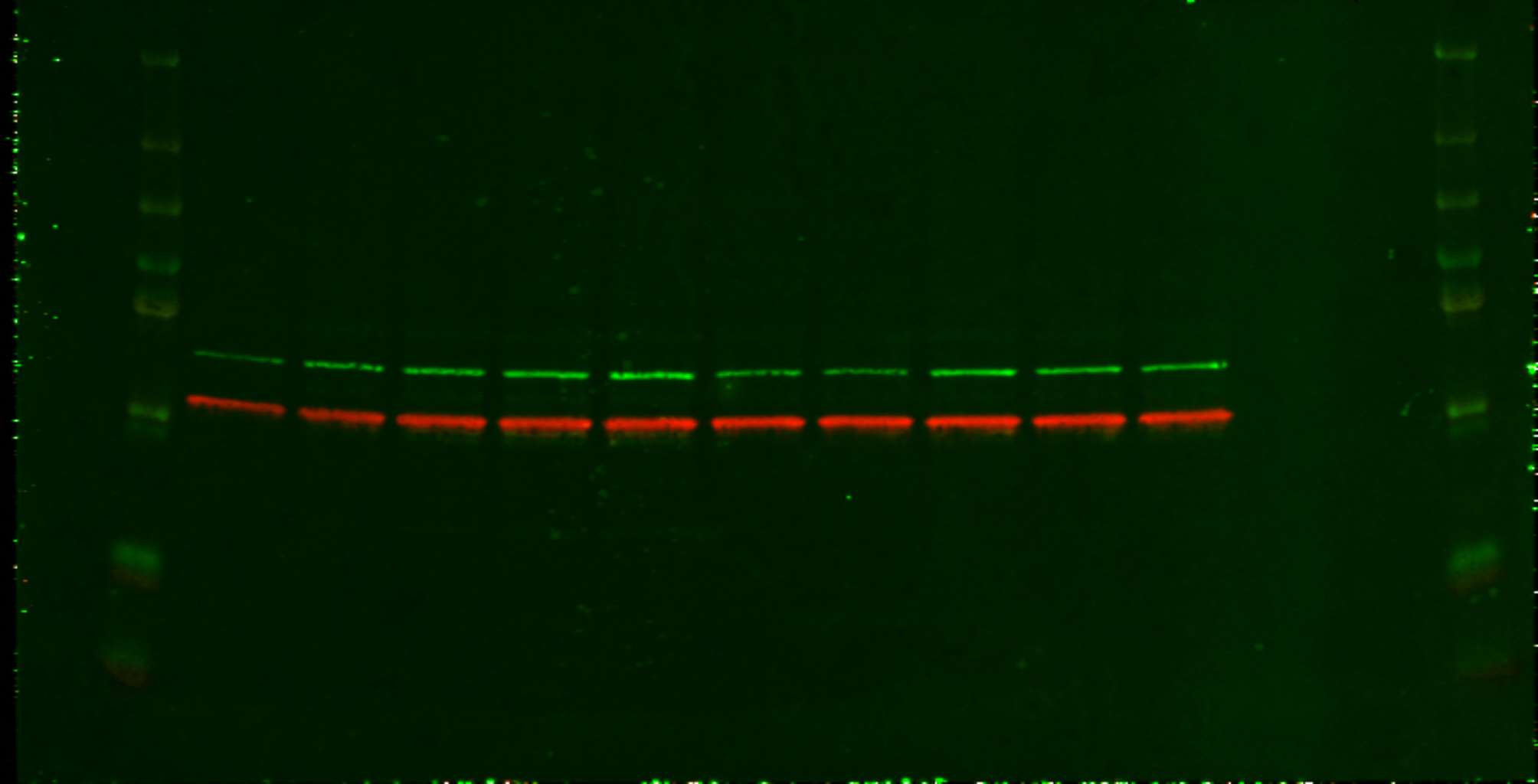

Supplement: S1 File — (TIF) [file pbio.3003153.s005.tif]
