# Supplementary material for: Brief early-life motor training induces behavioral changes and alters neuromuscular development in mice
Source: PLoS Biol. 2025 Apr 21;23(4):e3003153. doi: 10.1371/journal.pbio.3003153 (PMC12052215; doi:10.1371/journal.pbio.3003153)
Supplement: S1 Table — Over expressed genes are in blue. Under expressed genes are in Red. (DOCX) [file pbio.3003153.s004.docx]

| **geneID** | **logFC** | **PValue** | **Description** |
| --- | --- | --- | --- |
| Atp13a2 | -1.31 | 1.1E-06 | ATPase type 13A2 |
| Cdh22 | -2.35 | 1.2E-06 | cadherin 22 |
| Gm1943 | -2.46 | 1.5E-06 | predicted gene 1943 |
| Rtn2 | -1.38 | 2.4E-06 | reticulon 2 (Z-band associated protein) |
| Fam134a | -1.04 | 3.1E-06 | None |
| Zdhhc7 | -1.25 | 4.7E-06 | zinc finger, DHHC domain containing 7 |
| Sema6b | -1.75 | 6.7E-06 | sema domain, transmembrane domain (TM), and cytoplasmic domain, (semaphorin) 6B |
| Rab24 | -1.41 | 7.1E-06 | RAB24, member RAS oncogene family |
| Ecel1 | -1.18 | 7.3E-06 | endothelin converting enzyme-like 1 |
| Bag1 | -1.94 | 7.5E-06 | BCL2-associated athanogene 1 |
| Pink1 | -1.18 | 8.8E-06 | PTEN induced putative kinase 1 |
| Glrx5 | -1.94 | 9.8E-06 | glutaredoxin 5 |
| Fbxo31 | -1.40 | 1.1E-05 | F-box protein 31 |
| Prr24 | -1.74 | 1.3E-05 | None |
| Tysnd1 | -1.70 | 1.6E-05 | trypsin domain containing 1 |
| Begain | -1.61 | 1.7E-05 | brain-enriched guanylate kinase-associated |
| Mcat | -1.49 | 1.8E-05 | malonyl CoA:ACP acyltransferase (mitochondrial) |
| Ptges2 | -1.35 | 1.9E-05 | prostaglandin E synthase 2 |
| Mxra7 | -1.64 | 2.0E-05 | matrix-remodelling associated 7 |
| Tmem8 | -1.63 | 2.0E-05 | None |
| Syndig1l | -1.41 | 2.0E-05 | synapse differentiation inducing 1 like |
| Galnt9 | -1.18 | 2.1E-05 | polypeptide N-acetylgalactosaminyltransferase 9 |
| Ret | -1.10 | 2.1E-05 | ret proto-oncogene |
| Lrp11 | -1.16 | 2.4E-05 | low density lipoprotein receptor-related protein 11 |
| Scx | -1.88 | 2.5E-05 | scleraxis scleraxis bHLH transcription factor |
| Smyd2 | -1.03 | 2.6E-05 | SET and MYND domain containing 2 |
| Prrt3 | -1.41 | 2.8E-05 | proline-rich transmembrane protein 3 |
| Cdk16 | -1.38 | 3.0E-05 | cyclin dependent kinase 16 |
| Gal3st3 | -1.83 | 3.1E-05 | galactose-3-O-sulfotransferase 3 |
| Pithd1 | -1.36 | 3.2E-05 | PITH (C-terminal proteasome-interacting domain of thioredoxin-like) domain containing 1 |
| Galnt10 | -1.20 | 3.2E-05 | polypeptide N-acetylgalactosaminyltransferase 10 |
| Fam43b | -1.62 | 3.3E-05 | family with sequence similarity 43, member B |
| Isoc1 | -1.02 | 3.4E-05 | isochorismatase domain containing 1 |
| B4galt2 | -1.13 | 3.7E-05 | UDP-Gal:betaGlcNAc beta 1,4- galactosyltransferase, polypeptide 2 |
| St3gal3 | -1.07 | 3.9E-05 | ST3 beta-galactoside alpha-2,3-sialyltransferase 3 |
| Tmem248 | -1.13 | 3.9E-05 | transmembrane protein 248 |
| Chpf | -1.10 | 4.3E-05 | chondroitin polymerizing factor |
| Map1lc3a | -1.19 | 4.4E-05 | microtubule-associated protein 1 light chain 3 alpha |
| Gm4980 | -1.53 | 4.9E-05 | None |
| Get4 | -1.53 | 4.9E-05 | golgi to ER traffic protein 4 |
| Cited4 | -1.99 | 5.0E-05 | Cbp/p300-interacting transactivator, with Glu/Asp-rich carboxy-terminal domain, 4 |
| Mtch1 | -1.10 | 5.1E-05 | mitochondrial carrier 1 |
| Tmem55b | -1.33 | 5.5E-05 | None |
| Panx1 | -1.04 | 5.8E-05 | pannexin 1 |
| Vstm2l | -1.93 | 7.5E-05 | V-set and transmembrane domain containing 2-like |
| Smarcd3 | -1.17 | 7.6E-05 | SWI/SNF related, matrix associated, actin dependent regulator of chromatin, subfamily d, member 3 |
| Nrtn | -2.73 | 7.6E-05 | neurturin |
| Polr2m | -1.00 | 8.0E-05 | polymerase (RNA) II (DNA directed) polypeptide M |
| Tmem160 | -1.48 | 8.1E-05 | transmembrane protein 160 |
| Klhl17 | -1.07 | 8.4E-05 | kelch-like 17 |
| Wbscr16 | -1.05 | 8.7E-05 | None |
| Btbd17 | -1.75 | 8.7E-05 | BTB (POZ) domain containing 17 |
| Noc2l | -1.25 | 9.4E-05 | NOC2 like nucleolar associated transcriptional repressor |
| Zfp771 | -2.89 | 9.8E-05 | zinc finger protein 771 |
| Rnf187 | -1.51 | 1.0E-04 | ring finger protein 187 |
| F8a | -1.04 | 1.1E-04 | factor 8-associated gene A |
| Rhbdl3 | -1.18 | 1.1E-04 | rhomboid like 3 |
| Odc1 | -1.51 | 1.2E-04 | ornithine decarboxylase, structural 1 |
| Zfp622 | -1.21 | 1.3E-04 | zinc finger protein 622 |
| Endog | -1.62 | 1.3E-04 | endonuclease G |
| Enho | -1.04 | 1.4E-04 | energy homeostasis associated |
| Slc9a3r2 | -1.00 | 1.5E-04 | None |
| Pgp | -1.43 | 1.5E-04 | phosphoglycolate phosphatase |
| Htra1 | -1.42 | 1.5E-04 | HtrA serine peptidase 1 |
| Rgmb | -1.78 | 1.5E-04 | repulsive guidance molecule family member B |
| Plbd2 | -1.03 | 1.6E-04 | phospholipase B domain containing 2 |
| Sema6c | -1.92 | 1.6E-04 | sema domain, transmembrane domain (TM), and cytoplasmic domain, (semaphorin) 6C |
| Cdh24 | -1.23 | 1.6E-04 | cadherin-like 24 |
| B3galt6 | -1.25 | 1.6E-04 | UDP-Gal:betaGal beta 1,3-galactosyltransferase, polypeptide 6 |
| Ttc9 | -1.43 | 1.7E-04 | tetratricopeptide repeat domain 9 |
| Hhipl1 | -1.44 | 1.7E-04 | hedgehog interacting protein-like 1 |
| Cmas | -1.09 | 1.7E-04 | cytidine monophospho-N-acetylneuraminic acid synthetase |
| Dtx1 | -1.00 | 1.8E-04 | deltex 1, E3 ubiquitin ligase |
| Podxl2 | -2.02 | 1.8E-04 | podocalyxin-like 2 |
| Wsb2 | -1.00 | 1.9E-04 | WD repeat and SOCS box-containing 2 |
| Pank2 | -1.21 | 1.9E-04 | pantothenate kinase 2 |
| Tmed2 | -1.23 | 2.1E-04 | transmembrane p24 trafficking protein 2 |
| Fzd9 | -1.61 | 2.2E-04 | frizzled class receptor 9 |
| Fam174b | -1.07 | 2.2E-04 | family with sequence similarity 174, member B |
| Rtn4rl2 | -3.91 | 2.2E-04 | reticulon 4 receptor-like 2 |
| Habp4 | -1.47 | 2.3E-04 | hyaluronic acid binding protein 4 |
| Tmem121 | -1.52 | 2.3E-04 | transmembrane protein 121 |
| Bod1 | -1.29 | 2.5E-04 | biorientation of chromosomes in cell division 1 |
| Igfbp2 | -1.67 | 2.5E-04 | insulin-like growth factor binding protein 2 |
| Gpr137c | -1.30 | 2.5E-04 | G protein-coupled receptor 137C |
| Camkv | -1.02 | 2.6E-04 | CaM kinase-like vesicle-associated |
| Plcg1 | -1.35 | 2.6E-04 | phospholipase C, gamma 1 |
| Lor | -1.26 | 2.6E-04 | loricrin cornified envelope precursor protein |
| Kcnj12 | -1.11 | 2.7E-04 | potassium inwardly-rectifying channel, subfamily J, member 12 |
| Cdr2l | -1.04 | 2.8E-04 | cerebellar degeneration-related protein 2-like |
| E130012A19Rik | -1.86 | 2.8E-04 | None |
| Rfxap | -1.24 | 2.9E-04 | regulatory factor X-associated protein |
| Plekhh3 | -2.13 | 2.9E-04 | pleckstrin homology domain containing, family H (with MyTH4 domain) member 3 |
| Them6 | -1.54 | 3.1E-04 | thioesterase superfamily member 6 |
| Hras1 | -1.13 | 3.1E-04 | None |
| Slc25a33 | -1.37 | 3.3E-04 | solute carrier family 25, member 33 |
| Kcnk12 | -2.77 | 3.3E-04 | potassium channel, subfamily K, member 12 |
| Iqsec2 | -1.39 | 3.5E-04 | IQ motif and Sec7 domain 2 |
| 1110008F13Rik | -2.24 | 3.5E-04 | None |
| Chic2 | -1.45 | 3.5E-04 | cysteine-rich hydrophobic domain 2 |
| Fam171a2 | -1.72 | 3.6E-04 | family with sequence similarity 171, member A2 |
| Nptxr | -4.46 | 3.7E-04 | neuronal pentraxin receptor |
| Obsl1 | -1.02 | 3.7E-04 | obscurin-like 1 |
| Yars2 | -1.12 | 3.8E-04 | tyrosyl-tRNA synthetase 2 (mitochondrial) |
| Grik5 | -1.84 | 3.9E-04 | glutamate receptor, ionotropic, kainate 5 (gamma 2) |
| Nos1ap | -1.32 | 4.1E-04 | nitric oxide synthase 1 (neuronal) adaptor protein |
| Ptov1 | -1.34 | 4.1E-04 | prostate tumor over expressed gene 1 |
| Kcnc3 | -1.05 | 4.2E-04 | potassium voltage gated channel, Shaw-related subfamily, member 3 |
| 1300018J18Rik | -1.86 | 4.5E-04 | None |
| Fndc4 | -1.02 | 4.7E-04 | fibronectin type III domain containing 4 |
| Osbpl10 | -1.50 | 5.1E-04 | oxysterol binding protein-like 10 |
| Adcy3 | -1.03 | 5.1E-04 | adenylate cyclase 3 |
| Tmem110 | -1.02 | 5.2E-04 | None |
| Sox4 | -1.18 | 5.5E-04 | SRY (sex determining region Y)-box 4 |
| AI848285 | -1.98 | 5.6E-04 | None |
| Bloc1s4 | -1.18 | 5.8E-04 | biogenesis of lysosomal organelles complex-1, subunit 4, cappuccino |
| Map3k10 | -1.09 | 5.9E-04 | mitogen-activated protein kinase kinase kinase 10 |
| Cabp7 | -1.48 | 6.2E-04 | calcium binding protein 7 |
| Tmem238 | -1.88 | 6.3E-04 | transmembrane protein 238 |
| Rnf11 | -1.23 | 6.5E-04 | ring finger protein 11 |
| Jund | -1.85 | 6.5E-04 | jun D proto-oncogene |
| Plxdc1 | -1.45 | 7.1E-04 | plexin domain containing 1 |
| Tspan33 | -1.19 | 7.2E-04 | tetraspanin 33 |
| Orai1 | -1.41 | 7.3E-04 | ORAI calcium release-activated calcium modulator 1 |
| Sntb2 | -1.35 | 7.5E-04 | syntrophin, basic 2 |
| Ddi2 | -1.55 | 7.8E-04 | DNA-damage inducible protein 2 |
| Taf10 | -1.79 | 8.1E-04 | TATA-box binding protein associated factor 10 |
| Arhgap42 | -1.00 | 8.1E-04 | Rho GTPase activating protein 42 |
| Smarcd2 | -1.05 | 8.4E-04 | SWI/SNF related, matrix associated, actin dependent regulator of chromatin, subfamily d, member 2 |
| Nr2f6 | -1.21 | 8.4E-04 | nuclear receptor subfamily 2, group F, member 6 |
| Zxdc | -1.13 | 8.6E-04 | ZXD family zinc finger C |
| Mrps30 | -1.06 | 9.3E-04 | mitochondrial ribosomal protein S30 |
| Gfra4 | -1.01 | 9.9E-04 | glial cell line derived neurotrophic factor family receptor alpha 4 |
| Rab11fip4 | -1.29 | 9.9E-04 | RAB11 family interacting protein 4 (class II) |
| Eya2 | -1.01 | 1.0E-03 | EYA transcriptional coactivator and phosphatase 2 |
| Hcn2 | -1.32 | 1.0E-03 | hyperpolarization-activated, cyclic nucleotide-gated K+ 2 |
| Ing2 | -1.19 | 1.1E-03 | inhibitor of growth family, member 2 |
| Taf5 | -1.23 | 1.2E-03 | TATA-box binding protein associated factor 5 |
| Fance | -1.07 | 1.2E-03 | Fanconi anemia, complementation group E |
| Anks6 | -1.25 | 1.3E-03 | ankyrin repeat and sterile alpha motif domain containing 6 |
| Mafa | -2.72 | 1.3E-03 | MAF bZIP transcription factor A |
| Coq2 | -1.14 | 1.3E-03 | coenzyme Q2 4-hydroxybenzoate polyprenyltransferase |
| Ube2j2 | -1.61 | 1.3E-03 | ubiquitin-conjugating enzyme E2J 2 |
| Ankrd13c | -1.16 | 1.4E-03 | ankyrin repeat domain 13c |
| 2810408M09Rik | -1.02 | 1.4E-03 | None |
| E2f1 | -1.04 | 1.4E-03 | E2F transcription factor 1 |
| Bloc1s3 | -1.46 | 1.4E-03 | biogenesis of lysosomal organelles complex-1, subunit 3 |
| Dusp5 | -3.56 | 1.4E-03 | dual specificity phosphatase 5 |
| Aven | -1.21 | 1.4E-03 | apoptosis, caspase activation inhibitor |
| Cdk5r2 | -1.72 | 1.4E-03 | cyclin dependent kinase 5, regulatory subunit 2 (p39) |
| Scn1b | -1.44 | 1.5E-03 | sodium channel, voltage-gated, type I, beta |
| Kcna3 | -1.65 | 1.6E-03 | potassium voltage-gated channel, shaker-related subfamily, member 3 |
| Socs1 | -1.26 | 1.6E-03 | suppressor of cytokine signaling 1 |
| Lama5 | -1.46 | 1.8E-03 | laminin, alpha 5 |
| Brf1 | -1.01 | 1.8E-03 | BRF1, RNA polymerase III transcription initiation factor 90 kDa subunit |
| Shisa2 | -1.25 | 1.8E-03 | shisa family member 2 |
| Eef1a2 | -1.12 | 1.9E-03 | eukaryotic translation elongation factor 1 alpha 2 |
| Otud3 | -1.86 | 2.2E-03 | OTU domain containing 3 |
| Cdv3 | -1.03 | 2.3E-03 | carnitine deficiency-associated gene expressed in ventricle 3 |
| Ccdc87 | -2.21 | 2.4E-03 | coiled-coil domain containing 87 |
| Trp53rk | -1.17 | 2.4E-03 | None |
| Frat2 | -1.41 | 2.4E-03 | frequently rearranged in advanced T cell lymphomas 2 |
| Rhpn1 | -1.15 | 2.4E-03 | rhophilin, Rho GTPase binding protein 1 |
| Heg1 | -1.17 | 2.5E-03 | heart development protein with EGF-like domains 1 |
| Uhmk1 | -1.90 | 2.6E-03 | U2AF homology motif (UHM) kinase 1 |
| Fam98c | -1.21 | 2.6E-03 | family with sequence similarity 98, member C |
| Pkn1 | -1.15 | 2.7E-03 | protein kinase N1 |
| Mrs2 | -1.92 | 2.7E-03 | MRS2 magnesium transporter |
| Alyref | -1.19 | 2.8E-03 | Aly/REF export factor |
| Dbp | -1.71 | 2.8E-03 | D site albumin promoter binding protein |
| Gal3st1 | -1.20 | 2.9E-03 | galactose-3-O-sulfotransferase 1 |
| Adra1d | -1.03 | 2.9E-03 | adrenergic receptor, alpha 1d |
| Rassf3 | -1.14 | 3.1E-03 | Ras association (RalGDS/AF-6) domain family member 3 |
| Gjc2 | -2.02 | 3.1E-03 | gap junction protein, gamma 2 |
| Sptbn4 | -1.22 | 3.1E-03 | spectrin beta, non-erythrocytic 4 |
| Gas2l2 | -1.55 | 3.4E-03 | growth arrest-specific 2 like 2 |
| C2cd4d | -3.45 | 3.5E-03 | C2 calcium-dependent domain containing 4D |
| Ank1 | -1.06 | 3.5E-03 | ankyrin 1, erythroid |
| Xylt1 | -1.27 | 3.7E-03 | xylosyltransferase 1 |
| Dexi | -1.15 | 3.7E-03 | dexamethasone-induced transcript |
| Fam172a | -1.04 | 3.8E-03 | family with sequence similarity 172, member A |
| Atp7b | -1.83 | 3.9E-03 | ATPase, Cu++ transporting, beta polypeptide |
| Pptc7 | -1.03 | 4.1E-03 | PTC7 protein phosphatase homolog |
| BC005561 | -1.11 | 4.2E-03 | None |
| C1ql1 | -1.52 | 4.2E-03 | complement component 1, q subcomponent-like 1 |
| Zfp36l2 | -1.14 | 4.2E-03 | zinc finger protein 36, C3H type-like 2 |
| Asb4 | -1.48 | 4.3E-03 | ankyrin repeat and SOCS box-containing 4 |
| Celsr1 | -1.86 | 4.4E-03 | cadherin, EGF LAG seven-pass G-type receptor 1 |
| Arhgap36 | -1.15 | 4.5E-03 | Rho GTPase activating protein 36 |
| Gp5 | -1.46 | 4.6E-03 | glycoprotein 5 (platelet) |
| Fam105b | -1.07 | 5.0E-03 | None |
| Glis3 | -3.19 | 5.0E-03 | GLIS family zinc finger 3 |
| Hmga1 | -1.90 | 5.1E-03 | high mobility group AT-hook 1 |
| Ntng2 | -1.11 | 5.4E-03 | netrin G2 |
| Macrod1 | -1.35 | 5.5E-03 | mono-ADP ribosylhydrolase 1 |
| 0610012G03Rik | -1.00 | 5.8E-03 | None |
| Mfsd12 | -1.28 | 5.9E-03 | major facilitator superfamily domain containing 12 |
| Lrfn1 | -1.02 | 5.9E-03 | leucine rich repeat and fibronectin type III domain containing 1 |
| H1fx | -1.12 | 6.2E-03 | None |
| Map3k3 | -1.06 | 6.5E-03 | mitogen-activated protein kinase kinase kinase 3 |
| Dyrk2 | -1.00 | 7.1E-03 | dual-specificity tyrosine-(Y)-phosphorylation regulated kinase 2 |
| Tagap | -1.51 | 7.4E-03 | T cell activation Rho GTPase activating protein |
| Notch4 | -1.27 | 7.8E-03 | notch 4 |
| Espn | -1.65 | 8.2E-03 | espin |
| Mocs3 | -1.40 | 8.4E-03 | molybdenum cofactor synthesis 3 |
| 3110021N24Rik | -1.13 | 8.7E-03 | None |
| Myh7 | -2.35 | 9.4E-03 | myosin, heavy polypeptide 7, cardiac muscle, beta |
| Fam69c | -1.12 | 9.4E-03 | None |
| Foxq1 | -1.24 | 9.6E-03 | forkhead box Q1 |
| Tigd5 | -1.11 | 1.0E-02 | tigger transposable element derived 5 |
| Scand1 | -1.17 | 1.0E-02 | SCAN domain-containing 1 |
| Tbx1 | -2.61 | 1.1E-02 | T-box 1 |
| Kcng4 | -1.29 | 1.1E-02 | potassium voltage-gated channel, subfamily G, member 4 |
| Ern1 | -1.07 | 1.1E-02 | endoplasmic reticulum (ER) to nucleus signalling 1 |
| 8030462N17Rik | -1.87 | 1.2E-02 | arkadia (RNF111) N-terminal like PKA signaling regulator 2N |
| Colq | -2.51 | 1.2E-02 | collagen-like tail subunit (single strand of homotrimer) of asymmetric acetylcholinesterase |
| 9930012K11Rik | -1.08 | 1.2E-02 | RIKEN cDNA 9930012K11 gene |
| Kcne3 | -2.83 | 1.3E-02 | potassium voltage-gated channel, Isk-related subfamily, gene 3 |
| Dnajc25 | -1.17 | 1.3E-02 | DnaJ heat shock protein family (Hsp40) member C25 |
| Tbx18 | -2.04 | 1.4E-02 | T-box18 |
| She | -1.42 | 1.4E-02 | src homology 2 domain-containing transforming protein E |
| Gpr27 | -1.43 | 1.6E-02 | G protein-coupled receptor 27 |
| Gc | -3.17 | 1.6E-02 | vitamin D binding protein |
| Col6a1 | -1.20 | 1.6E-02 | collagen, type VI, alpha 1 |
| Ano8 | -1.45 | 1.7E-02 | anoctamin 8 |
| Cckar | -2.65 | 1.7E-02 | cholecystokinin A receptor |
| Gpr62 | -1.86 | 1.7E-02 | G protein-coupled receptor 62 |
| Dapk3 | -1.22 | 1.7E-02 | death-associated protein kinase 3 |
| Yrdc | -1.12 | 1.8E-02 | yrdC domain containing (E.coli) |
| Piwil2 | -2.56 | 1.9E-02 | piwi-like RNA-mediated gene silencing 2 |
| Gypa | -2.67 | 1.9E-02 | glycophorin A |
| Piezo1 | -1.92 | 1.9E-02 | piezo-type mechanosensitive ion channel component 1 |
| Pkd2l1 | -2.56 | 1.9E-02 | polycystic kidney disease 2-like 1 |
| Hs6st3 | -1.36 | 2.0E-02 | heparan sulfate 6-O-sulfotransferase 3 |
| Fndc8 | -2.23 | 2.0E-02 | fibronectin type III domain containing 8 |
| Spef2 | -2.71 | 2.1E-02 | sperm flagellar 2 |
| 2310014L17Rik | -1.21 | 2.1E-02 | None |
| Zc3hav1 | -1.94 | 2.1E-02 | zinc finger CCCH type, antiviral 1 |
| Gm7694 | -1.17 | 2.2E-02 | predicted gene 7694 |
| Loxl4 | -1.49 | 2.2E-02 | lysyl oxidase-like 4 |
| E430018J23Rik | -1.14 | 2.2E-02 | RIKEN cDNA E430018J23 gene |
| Klrg2 | -1.18 | 2.2E-02 | killer cell lectin-like receptor subfamily G, member 2 |
| Igfbp7 | -1.19 | 2.2E-02 | insulin-like growth factor binding protein 7 |
| Spata5l1 | -1.80 | 2.2E-02 | AFG2 AAA ATPase homolog B |
| Gm16897 | -1.29 | 2.3E-02 | predicted gene, 16897 |
| Ajuba | -1.00 | 2.3E-02 | ajuba LIM protein |
| Sstr3 | -1.14 | 2.3E-02 | somatostatin receptor 3 |
| 4931440F15Rik | -1.61 | 2.4E-02 | None |
| Kcnh3 | -2.09 | 2.4E-02 | potassium voltage-gated channel, subfamily H (eag-related), member 3 |
| Ppp1r3e | -1.48 | 2.5E-02 | protein phosphatase 1, regulatory subunit 3E |
| Adamts6 | -1.25 | 2.5E-02 | a disintegrin-like and metallopeptidase (reprolysin type) with thrombospondin type 1 motif, 6 |
| Grasp | -1.11 | 2.6E-02 | None |
| Ankle1 | -1.07 | 2.6E-02 | ankyrin repeat and LEM domain containing 1 |
| Gm711 | -2.02 | 2.6E-02 | None |
| Chdh | -1.85 | 2.7E-02 | choline dehydrogenase |
| 1700018L02Rik | -1.79 | 2.7E-02 | RIKEN cDNA 1700018L02 gene |
| Il20ra | -1.69 | 2.8E-02 | interleukin 20 receptor, alpha |
| Gdf5 | -1.12 | 2.8E-02 | growth differentiation factor 5 |
| Obscn | -1.07 | 2.8E-02 | obscurin, cytoskeletal calmodulin and titin-interacting RhoGEF |
| Ttc16 | -2.59 | 2.9E-02 | tetratricopeptide repeat domain 16 |
| Oaf | -1.22 | 2.9E-02 | out at first homolog |
| Npas4 | -1.82 | 3.0E-02 | neuronal PAS domain protein 4 |
| Pcdhgb2 | -1.73 | 3.0E-02 | protocadherin gamma subfamily B, 2 |
| Oas1c | -1.00 | 3.1E-02 | 2'-5' oligoadenylate synthetase 1C |
| Ccdc3 | -1.22 | 3.1E-02 | coiled-coil domain containing 3 |
| C78339 | -1.35 | 3.1E-02 | None |
| Trpv4 | -1.46 | 3.2E-02 | transient receptor potential cation channel, subfamily V, member 4 |
| Myo1h | -1.72 | 3.2E-02 | myosin 1H |
| Scube3 | -1.05 | 3.3E-02 | signal peptide, CUB domain, EGF-like 3 |
| Rgag1 | -1.22 | 3.3E-02 | None |
| Atp2a3 | -1.05 | 3.5E-02 | ATPase, Ca++ transporting, ubiquitous |
| Ggt5 | -1.14 | 3.5E-02 | gamma-glutamyltransferase 5 |
| Emilin2 | -1.92 | 3.5E-02 | elastin microfibril interfacer 2 |
| Mrvi1 | -1.48 | 3.6E-02 | None |
| Mybpc1 | -1.62 | 3.7E-02 | myosin binding protein C, slow-type |
| Stab1 | -1.08 | 3.9E-02 | stabilin 1 |
| Tmem154 | -1.35 | 4.1E-02 | transmembrane protein 154 |
| Ddx4 | -1.66 | 4.1E-02 | DEAD box helicase 4 |
| Sned1 | -1.53 | 4.1E-02 | sushi, nidogen and EGF-like domains 1 |
| Dnahc10 | -1.33 | 4.2E-02 | None |
| Cebpd | -1.06 | 4.2E-02 | CCAAT/enhancer binding protein (C/EBP), delta |
| Apol6 | -1.37 | 4.3E-02 | apolipoprotein L 6 |
| Pawr | -1.71 | 4.4E-02 | PRKC, apoptosis, WT1, regulator |
| Col4a6 | -2.09 | 4.5E-02 | collagen, type IV, alpha 6 |
| Asprv1 | -1.79 | 4.5E-02 | aspartic peptidase, retroviral-like 1 |
| Cd7 | -2.17 | 4.6E-02 | CD7 antigen |
| E030024N20Rik | -1.17 | 4.7E-02 | None |
| Mycbpap | -1.38 | 4.8E-02 | MYCBP associated protein |
| Nlrc3 | -2.04 | 4.9E-02 | NLR family, CARD domain containing 3 |
| Gm6787 | -1.66 | 4.9E-02 | predicted gene 6787 |
| Klf14 | -1.06 | 4.9E-02 | Kruppel-like transcription factor 14 |
| Rab37 | -1.16 | 5.0E-02 | RAB37, member RAS oncogene family |
| Id3 | 1.27 | 3.9E-06 | inhibitor of DNA binding 3 |
| Cyb5 | 1.07 | 2.0E-05 | None |
| Gng5 | 1.08 | 2.2E-05 | G protein subunit gamma 5 |
| Id2 | 1.20 | 2.6E-05 | inhibitor of DNA binding 2 |
| Serf1 | 1.13 | 3.1E-05 | small EDRK-rich factor 1 |
| Myc | 1.16 | 3.2E-05 | myelocytomatosis oncogene |
| Uqcrb | 1.00 | 3.9E-05 | ubiquinol-cytochrome c reductase binding protein |
| Hopx | 1.06 | 4.1E-05 | HOP homeobox |
| Pfdn4 | 1.00 | 4.4E-05 | prefoldin 4 |
| Mt1 | 1.11 | 5.4E-05 | metallothionein 1 |
| Ptma | 1.04 | 7.2E-05 | prothymosin alpha |
| Naa38 | 1.12 | 8.0E-05 | N(alpha)-acetyltransferase 38, NatC auxiliary subunit |
| Wbp5 | 1.01 | 1.0E-04 | None |
| Ifitm2 | 1.27 | 1.0E-04 | interferon induced transmembrane protein 2 |
| 1110059G10Rik | 1.19 | 1.1E-04 | RIKEN cDNA 1110059G10 gene |
| Gpx8 | 1.15 | 1.2E-04 | glutathione peroxidase 8 (putative) |
| BC064078 | 1.11 | 1.4E-04 | cDNA sequence BC064078 |
| Tmem100 | 1.00 | 1.4E-04 | transmembrane protein 100 |
| Krt10 | 1.13 | 1.8E-04 | keratin 10 |
| Neurod6 | 5.82 | 2.0E-04 | neurogenic differentiation 6 |
| Snhg3 | 1.00 | 2.3E-04 | small nucleolar RNA host gene 3 |
| S100a11 | 1.36 | 2.6E-04 | S100 calcium binding protein A11 |
| Hmgb2 | 1.08 | 4.0E-04 | high mobility group box 2 |
| Spa17 | 1.12 | 4.2E-04 | sperm autoantigenic protein 17 |
| S100a6 | 1.16 | 4.3E-04 | S100 calcium binding protein A6 (calcyclin) |
| Cks2 | 1.15 | 5.0E-04 | CDC28 protein kinase regulatory subunit 2 |
| BC089491 | 1.05 | 5.1E-04 | selenoprotein V |
| Bex4 | 1.01 | 5.5E-04 | brain expressed X-linked 4 |
| Id1 | 1.02 | 5.6E-04 | inhibitor of DNA binding 1, HLH protein |
| Hoxb6 | 1.19 | 6.6E-04 | homeobox B6 |
| Arl6ip6 | 1.06 | 7.5E-04 | ADP-ribosylation factor-like 6 interacting protein 6 |
| Gad2 | 1.01 | 8.2E-04 | glutamic acid decarboxylase 2 |
| Gpr6 | 2.04 | 8.7E-04 | G protein-coupled receptor 6 |
| Rhcg | 1.24 | 8.9E-04 | Rhesus blood group-associated C glycoprotein |
| Sp8 | 2.06 | 9.4E-04 | trans-acting transcription factor 8 |
| Apod | 1.46 | 9.5E-04 | apolipoprotein D |
| Cenpq | 1.03 | 1.0E-03 | centromere protein Q |
| 1110006O24Rik | 1.35 | 1.2E-03 | RIKEN cDNA 1110006O24 gene |
| Mab21l2 | 1.57 | 1.3E-03 | mab-21-like 2 |
| Cdkn1a | 1.10 | 1.4E-03 | cyclin dependent kinase inhibitor 1A (P21) |
| Crip1 | 1.43 | 1.4E-03 | cysteine-rich protein 1 (intestinal) |
| Hoxb7 | 1.08 | 1.5E-03 | homeobox B7 |
| Blnk | 2.94 | 1.6E-03 | B cell linker |
| 2810408I11Rik | 1.99 | 1.7E-03 | RIKEN cDNA 2810408I11 gene |
| Lhx5 | 1.58 | 1.9E-03 | LIM homeobox protein 5 |
| Mbd2 | 1.14 | 2.1E-03 | methyl-CpG binding domain protein 2 |
| Npy | 1.47 | 2.3E-03 | neuropeptide Y |
| 0610007N19Rik | 1.29 | 2.3E-03 | None |
| Barhl2 | 3.64 | 2.3E-03 | BarH like homeobox 2 |
| Foxp2 | 1.32 | 2.7E-03 | forkhead box P2 |
| Dnajc28 | 1.02 | 3.2E-03 | DnaJ heat shock protein family (Hsp40) member C28 |
| Mex3c | 1.01 | 3.2E-03 | mex3 RNA binding family member C |
| Mia | 1.11 | 3.8E-03 | MIA SH3 domain containing |
| Plscr1 | 1.73 | 4.0E-03 | phospholipid scramblase 1 |
| Cpne4 | 1.10 | 4.1E-03 | copine IV |
| Mthfs | 1.15 | 4.2E-03 | 5, 10-methenyltetrahydrofolate synthetase |
| Crabp2 | 1.74 | 4.3E-03 | cellular retinoic acid binding protein II |
| Kremen2 | 1.26 | 4.4E-03 | kringle containing transmembrane protein 2 |
| Igsf9 | 1.69 | 4.7E-03 | immunoglobulin superfamily, member 9 |
| Hdx | 1.55 | 4.9E-03 | highly divergent homeobox |
| Gm14204 | 1.22 | 5.0E-03 | predicted gene 14204 |
| Hoxd11 | 1.20 | 5.2E-03 | homeobox D11 |
| 1700096K18Rik | 1.01 | 5.3E-03 | RIKEN cDNA 1700096K18 gene |
| B230209E15Rik | 1.25 | 5.8E-03 | RIKEN cDNA B230209E15 gene |
| Col3a1 | 3.08 | 5.8E-03 | collagen, type III, alpha 1 |
| Lekr1 | 1.03 | 6.0E-03 | leucine, glutamate and lysine rich 1 |
| Reg3b | 1.66 | 6.4E-03 | regenerating islet-derived 3 beta |
| Gmnn | 1.05 | 6.4E-03 | geminin |
| Ascl2 | 1.39 | 6.7E-03 | achaete-scute family bHLH transcription factor 2 |
| Ebf3 | 1.77 | 6.7E-03 | early B cell factor 3 |
| Casp6 | 1.15 | 7.2E-03 | caspase 6 |
| Hist2h3c1 | 2.32 | 7.2E-03 | None |
| Tcfl5 | 2.12 | 7.7E-03 | transcription factor-like 5 (basic helix-loop-helix) |
| Hfe2 | 2.43 | 7.8E-03 | None |
| Ttc30b | 1.01 | 8.0E-03 | intraflagellar transport 70B |
| 2010003O02Rik | 1.04 | 8.0E-03 | None |
| Onecut3 | 1.29 | 8.3E-03 | one cut domain, family member 3 |
| Col1a1 | 3.94 | 8.3E-03 | collagen, type I, alpha 1 |
| Faim3 | 1.35 | 8.3E-03 | None |
| 1500015O10Rik | 1.02 | 8.4E-03 | None |
| Tspo | 1.01 | 8.5E-03 | translocator protein |
| Oas1b | 3.36 | 8.5E-03 | 2'-5' oligoadenylate synthetase 1B |
| Apoa1 | 1.15 | 8.6E-03 | apolipoprotein A-I |
| Serpinf1 | 1.23 | 8.8E-03 | serine (or cysteine) peptidase inhibitor, clade F, member 1 |
| Fam43a | 1.19 | 8.9E-03 | family with sequence similarity 43, member A |
| Gm20063 | 1.28 | 9.4E-03 | predicted gene, 20063 |
| Tnfsf9 | 1.19 | 9.5E-03 | tumor necrosis factor (ligand) superfamily, member 9 |
| Nrn1 | 1.34 | 9.8E-03 | neuritin 1 |
| Nupr1 | 2.01 | 1.0E-02 | nuclear protein transcription regulator 1 |
| Dlx3 | 2.11 | 1.0E-02 | distal-less homeobox 3 |
| Fam122b | 1.10 | 1.1E-02 | None |
| Padi6 | 3.26 | 1.1E-02 | peptidyl arginine deiminase, type VI |
| Pon3 | 1.89 | 1.1E-02 | paraoxonase 3 |
| Troap | 1.35 | 1.2E-02 | trophinin associated protein |
| Cnksr3 | 1.17 | 1.2E-02 | Cnksr family member 3 |
| Kctd12b | 1.08 | 1.2E-02 | potassium channel tetramerisation domain containing 12b |
| 2610034M16Rik | 2.71 | 1.2E-02 | None |
| Ppp1r3g | 1.30 | 1.2E-02 | protein phosphatase 1, regulatory subunit 3G |
| 1110020A21Rik | 1.75 | 1.2E-02 | RIKEN cDNA 1110020A21 gene |
| Hhex | 1.59 | 1.3E-02 | hematopoietically expressed homeobox |
| Lpl | 1.39 | 1.3E-02 | lipoprotein lipase |
| Gm5468 | 1.44 | 1.3E-02 | predicted gene 5468 |
| Zfp133-ps | 1.06 | 1.3E-02 | zinc finger protein 133, pseudogene |
| 2310030G06Rik | 1.10 | 1.3E-02 | RIKEN cDNA 2310030G06 gene |
| Mex3b | 1.05 | 1.3E-02 | mex3 RNA binding family member B |
| Hoxc11 | 1.89 | 1.4E-02 | homeobox C11 |
| Prx | 3.11 | 1.4E-02 | periaxin |
| Vsx2 | 1.38 | 1.5E-02 | visual system homeobox 2 |
| Armc2 | 1.32 | 1.5E-02 | armadillo repeat containing 2 |
| 4931414P19Rik | 1.34 | 1.6E-02 | RIKEN cDNA 4931414P19 gene |
| Zfp820 | 1.01 | 1.6E-02 | zinc finger protein 820 |
| Upk3a | 2.01 | 1.6E-02 | uroplakin 3A |
| Hotair | 2.13 | 1.6E-02 | HOX transcript antisense RNA (non-protein coding) |
| Atf5 | 1.05 | 1.7E-02 | activating transcription factor 5 |
| Nrgn | 1.96 | 1.7E-02 | neurogranin |
| Wif1 | 1.92 | 1.8E-02 | Wnt inhibitory factor 1 |
| Scml2 | 1.35 | 2.0E-02 | Scm polycomb group protein like 2 |
| Hoxc5 | 1.15 | 2.0E-02 | homeobox C5 |
| Ticrr | 1.16 | 2.0E-02 | TOPBP1-interacting checkpoint and replication regulator |
| Gm11627 | 1.28 | 2.0E-02 | predicted gene 11627 |
| Cd52 | 1.43 | 2.1E-02 | CD52 antigen |
| Ptpn20 | 1.32 | 2.1E-02 | protein tyrosine phosphatase, non-receptor type 20 |
| Nr4a2 | 1.16 | 2.1E-02 | nuclear receptor subfamily 4, group A, member 2 |
| Barhl1 | 2.07 | 2.2E-02 | BarH like homeobox 1 |
| A630023A22Rik | 2.16 | 2.2E-02 | RIKEN cDNA A630023A22 gene |
| B330016D10Rik | 1.02 | 2.2E-02 | None |
| Zfp456 | 2.54 | 2.3E-02 | zinc finger protein 456 |
| Otogl | 2.40 | 2.4E-02 | otogelin-like |
| Edn2 | 1.09 | 2.4E-02 | endothelin 2 |
| Gm13031 | 1.94 | 2.5E-02 | predicted gene 13031 |
| Tifa | 1.36 | 2.5E-02 | TRAF-interacting protein with forkhead-associated domain |
| Spdl1 | 1.10 | 2.5E-02 | spindle apparatus coiled-coil protein 1 |
| Ppp1r3b | 1.50 | 2.6E-02 | protein phosphatase 1, regulatory subunit 3B |
| 4930479D17Rik | 1.98 | 2.6E-02 | RIKEN cDNA 4930479D17 gene |
| 1810014B01Rik | 1.02 | 2.7E-02 | RIKEN cDNA 1810014B01 gene |
| Mpp4 | 2.16 | 2.7E-02 | membrane protein, palmitoylated 4 (MAGUK p55 subfamily member 4) |
| Iapp | 2.94 | 2.8E-02 | islet amyloid polypeptide |
| Tbc1d8b | 1.66 | 2.8E-02 | TBC1 domain family, member 8B |
| Car13 | 1.93 | 3.0E-02 | carbonic anhydrase 13 |
| Trh | 1.84 | 3.0E-02 | thyrotropin releasing hormone |
| Otp | 1.48 | 3.0E-02 | orthopedia homeobox |
| Slfn5 | 1.10 | 3.1E-02 | schlafen 5 |
| 4931440P22Rik | 1.21 | 3.1E-02 | RIKEN cDNA 4931440P22 gene |
| Atp2a1 | 2.45 | 3.1E-02 | ATPase, Ca++ transporting, cardiac muscle, fast twitch 1 |
| Gm16702 | 1.26 | 3.2E-02 | None |
| Zfhx4 | 1.04 | 3.2E-02 | zinc finger homeodomain 4 |
| Cryba4 | 1.89 | 3.3E-02 | crystallin, beta A4 |
| Fut4 | 1.70 | 3.4E-02 | fucosyltransferase 4 |
| Pabpc4l | 1.29 | 3.5E-02 | poly(A) binding protein, cytoplasmic 4-like |
| Lama2 | 1.43 | 3.5E-02 | laminin, alpha 2 |
| AB099516 | 1.74 | 3.6E-02 | methyltransferase hypoxia inducible domain containing 1 |
| Spdef | 1.48 | 3.6E-02 | SAM pointed domain containing ets transcription factor |
| Cyp4f14 | 2.52 | 3.7E-02 | cytochrome P450, family 4, subfamily f, polypeptide 14 |
| Rapsn | 1.26 | 3.7E-02 | receptor-associated protein of the synapse |
| 1810011O10Rik | 1.91 | 3.7E-02 | None |
| Postn | 1.42 | 3.7E-02 | periostin, osteoblast specific factor |
| Fgd3 | 2.70 | 3.8E-02 | FYVE, RhoGEF and PH domain containing 3 |
| Gypc | 1.45 | 3.8E-02 | glycophorin C |
| Ctsw | 1.09 | 3.9E-02 | cathepsin W |
| Alox5 | 2.61 | 3.9E-02 | arachidonate 5-lipoxygenase |
| Gm13845 | 1.14 | 3.9E-02 | None |
| Zfp455 | 1.50 | 3.9E-02 | zinc finger protein 455 |
| Klri1 | 2.25 | 4.0E-02 | killer cell lectin-like receptor family I member 1 |
| Plekhg6 | 1.98 | 4.0E-02 | pleckstrin homology domain containing, family G (with RhoGef domain) member 6 |
| Tfap2b | 2.12 | 4.1E-02 | transcription factor AP-2 beta |
| Ccl2 | 1.43 | 4.1E-02 | chemokine (C-C motif) ligand 2 |
| Gm20754 | 1.36 | 4.2E-02 | predicted gene, 20754 |
| 9130008F23Rik | 1.30 | 4.2E-02 | RIKEN cDNA 9130008F23 gene |
| Arhgap8 | 1.56 | 4.2E-02 | Rho GTPase activating protein 8 |
| Ccdc63 | 1.52 | 4.3E-02 | coiled-coil domain containing 63 |
| Fstl3 | 1.78 | 4.3E-02 | follistatin-like 3 |
| Dkkl1 | 1.06 | 4.4E-02 | dickkopf-like 1 |
| E2f8 | 1.07 | 4.5E-02 | E2F transcription factor 8 |
| Tescl | 1.94 | 4.5E-02 | tescalcin-like |
| 2310069G16Rik | 1.03 | 4.5E-02 | None |
| Ly96 | 1.06 | 4.6E-02 | lymphocyte antigen 96 |
| Grp | 1.88 | 4.6E-02 | gastrin releasing peptide |
| Dmc1 | 1.81 | 4.6E-02 | DNA meiotic recombinase 1 |
| Fam107a | 1.44 | 4.7E-02 | family with sequence similarity 107, member A |
| Myl1 | 1.63 | 4.7E-02 | myosin, light polypeptide 1 |
| Ccdc27 | 1.48 | 4.8E-02 | coiled-coil domain containing 27 |
| Pcdhga5 | 1.67 | 4.8E-02 | protocadherin gamma subfamily A, 5 |
| Fam117a | 1.05 | 4.8E-02 | family with sequence similarity 117, member A |
| Akr1c19 | 1.30 | 4.9E-02 | aldo-keto reductase family 1, member C19 |
| Nfkbid | 1.18 | 4.9E-02 | nuclear factor of kappa light polypeptide gene enhancer in B cells inhibitor, delta |
| 4732415M23Rik | 1.04 | 4.9E-02 | None |

**Supplementary Table 1.** Differentially expressed genes between trained- and untrained lateral motor column of P3 mice. Over expressed genes are in blue. Under expressed genes are in Red.
