# Supplementary material for: Brief early-life motor training induces behavioral changes and alters neuromuscular development in mice
Source: PLoS Biol. 2025 Apr 21;23(4):e3003153. doi: 10.1371/journal.pbio.3003153 (PMC12052215; doi:10.1371/journal.pbio.3003153)
Supplement: Attachment — Submitted filename: Plos_Biol_Response to Reviewers(1).pdf [file pbio.3003153.s008.pdf]

The authors sincerely thank the reviewers and the editor for their time and effort in reviewing our manuscript and for providing a constructive report that undoubtedly improves the quality of our study. We have made every effort to address the comments as thoroughly as possible. The RNA-seq results section and the corresponding figure have been completely revised, and a table has been added to detail the differentially expressed genes between trained and untrained lateral motor columns. Additionally, we have provided one video to illustrate the swimming patterns of postnatal day 3 trained and untrained pups.
Our responses to the comments are provided below in blue. Edits made to the main text, figures, and tables in response to these comments are also highlighted in blue in the "track changes" version of our manuscript.

REVIEWS:
Reviewer #1: This study explored the influence of increased motor activity in newborn mice during early period of development of spinal cord circuitry and hindlimb muscles. The main conclusion is that a very short motor training performed just after birth is able to induce functional adaptation in the developing neuromuscular system that could persist several days. The study is impressive in the scope and a number of electrophysiological, morphological and biochemical analyses performed, and the work is very well done. In vitro recordings on spinal cord slices are challenging, and the authors amassed a convincing number of recordings on which they base their conclusions.
The study is implemented with the high degree of rigor and technical excellence. Analyses are carefully performed and presentation is clear in general. The results are definitely interesting and a bit surprising, considering a very short-lasting training sessions. I have no significant criticisms. However, I would advice the Authors to reconsider presentation of all the detailed data in the tables and figures. A number of figures and plots is overwhelming, and some of them repeat the same data already presented in tables 2 and 3. It is a question whether to show all the insignificant data, which are shortly mentioned in the Results, but not discussed further.

In agreement with the reviewer's comment, we have removed the AHP parameter values from Table 3, as these data are already presented in Figure 3A3–A6, and the *f*-I linear slope values from Table 4, as they are shown in Figure 3B2. Corresponding changes have also been made to the main text and figure legends.

The minor points which should be corrected or clarified:
- it is difficult to find a number of mice used in particular groups for analyses, a total number, division into trained and untrained groups; sometimes a number of mice is indicated in the text, but frequently it is not, and this makes more difficult to assess the importance of particular changes among a substantive number of data;

All sample numbers are now indicated in the figures or their corresponding legends. This information has been added where it was previously missing in the revised version of the manuscript.

- a number of samples in Table 5 as well as a number of recordings used for violin plots might also be indicated in the plots or in the legends to make them more informative; presentation of a percentage in charts is informative, but should also be supported with a number of neurons/samples (Figs. 1BC, 3C2, 4D, 10C, 11);

The number of samples has been added to the legend of Table 5. The number of neurons/samples present in percentage charts is indicated in the figure legends.

- several data in Table 3 and 4 need to be corrected (rheobase is presented in nA? AP rise time in ms? current max in pA?)

There were indeed mistakes concerning the units mentioned in Tables 3 and 4. The corrections have been made, and we have thoroughly checked the manuscript to ensure no other errors were present. We apologize for these confusing mistakes.

- Fig. 3A1: is the scale bars (50ms and 2000pA) real for the presented record? It is stated in methods that "AHP parameters were computed after eliciting a single action potential (AP), with a brief 7 ms, 0.25 nA depolarizing current pulse". Basing on the scale bars the pulse here seems to be 2.5nA and duration at least twice longer, the same doubt concerns the AP and AHP duration. It is difficult for a reader to check such details in each presented chart, but when such a mistake has been found once, I would encourage the Authors to check such and similar details throughout the paper and all figures.

The reviewer is correct, and there was indeed an error—not in the scale bars in Figure 3, but in the methods section. The text has been revised to: "AHP parameters were computed after eliciting a single action potential (AP) with a brief 7 ms, **2.5 nA** depolarizing current pulse". Regarding the duration, we believe the scale bar is correct, as 7 mm corresponds to 50 ms, with a pulse length of approximately 1 mm, resulting in a duration of around 7 ms. We have thoroughly checked all scale bars and unit values throughout the paper.

- I have concern about clear outliners visible in some of the violin plots showing distribution of data (especially in Fig. 3A5, Fig. 4 C2 and C3). Are the Authors sure that these measurements were based on reliable recordings, not suffering from an erratic observation or instability of a preparation? Could this be connected to slow-fast differences between MNs, and obviously, slow and fast MNs were not distinguished?

The data distributions we obtained concerning MN membrane properties are consistent with those reported in our previous studies (Tartas et al., FINC 2010; Quilgars et al., FICN 2021) and by other groups (for example: Durand et al., FINC 2015; Smith and Brownstone, J. Physiol. 2020). The study by Smith and Brownstone shows a similarly broad distribution of AHP parameters and sag slope at P2–P3. In our study, we did not differentiate fast and slow motoneurons, flexor-extensor or alpha-beta motoneurons. At P3, motoneuron differentiation into fast and slow subtypes is still incomplete, as muscle fibers remain undifferentiated and poly-innervated at this stage. Motoneuron profiles could have been predicted using immunohistochemistry or in situ hybridization (e.g., osteopontin, metalloproteinase 9, or

Dlk1). Their dendritic morphology also varies significantly at this developmental stage (Durand et al, Neuroscience 2012). Additionally, the flexor-extensor identity of motoneurons influences their maturation, with flexor motoneurons maturing faster than extensor ones. Thus, we think that the diversity of motoneurons within the lateral motor column combined to distinct maturation timelines, is reflected in the data distributions we obtained.

Reviewer #2: In this work, the authors designed a short motor training regimen at P1 and P2 to decode the impact of early training on the development of spinal motor neurons and hindlimb muscles using the combined techniques including behavioral analysis, RNA-seq, electrophysiological recordings, immunostaining, et al. They demonstrated that a short training caused many changes in newborn mice including swimming performances, gene expression, electrophysiological characteristics of spinal motor neurons, muscle development. The study is interesting, but the authors should strengthen the logical explanation of the changes they identified in different aspects.

I have some comments as below:

1. The author should give a brief explanation of the duration time designed in the training paradigm since that 7 sec or 15 sec were very short.

We had to establish a completely new training protocol adapted to newborn rodents as we were, to the best of our knowledge, the first to train P1-P2 mouse pups. This training was developed almost entirely empirically, based on our observations of pup behavior in water. When this project started, we specifically chose to train very young animals to assess changes in activity-dependent synaptic plasticity (see Discussion). Our observations indicated that 15 seconds in water is the threshold at which P1 pups began to exhibit signs of fatigue. As shown in Figure 1C, after the third 15-second session (S3 in Figure 1A), the number of animals using all four limbs decreased. Additionally, we observed some pups that failed to swim for the full 15-second period and others that began to sink after S3. Based on these findings, we decided to limit the session duration to 15 seconds and repeat it five times. At the beginning of the study, we were not very optimistic about the efficacy of the training due to the short duration of the sessions the pups could endure. However, the evolution of their swimming patterns, along with our initial recordings of synaptic plasticity and the observed switch in plasticity profiles, confirmed that despite its brevity, the training protocol is effective in inducing adaptation within the spinal network. In fact, it is so effective that we had to limit the swimming test for untrained pups to 7 seconds—otherwise, as soon as we increased the first trial session duration, they began adapting their swimming behavior by the second session (S2).

To address this point the following sentences were added lines 114-118 in the Material and Methods section: “This training protocol is based on empirical observations of pup behavior in water. We observed that swimming sessions of 15 seconds marked the threshold at which P1 pups began to exhibit signs of fatigue and that a first session longer than 7 seconds triggers adaptations in swimming patterns during the second one.”

2. In Fig. 1B1：why are the numbers of animals in different test batches different within the same group (untrained or trained)? In the untrained group, the numbers were 64, 66, 61, and 61 at the trial 1, 2, 3, and 4 respectively. In the trained group, they were 110, 99, 103, and 91 at the training 1, 2, 3, and 4 respectively. Why did the authors use such a large number of animals for behavioral screening?

As stated in the manuscript line 332: we have considered only well-defined swimming patterns for analysis, and animals performing mixed patterns during the 7 seconds of motor scoring were ignored. This explains why the number of animals included in the analysis for each training session differ.
The behavior of all trained pups used in the present study at P3 for electrophysiological recordings, transcriptomic analysis, HPLC analysis, and immunohistology was screened. This explains the large number of animals tested. There was a mistake in Fig. 1B, as the number of trained pups was not 110…, but 241…, as stated in Fig. 1C. The numbers present in the first version of Fig.1B for trained mice were only for one value of motor score (0 or 1) and did not correspond to the total of animals tested in one given training. We apologize for this mistake that has been corrected in the revised version of the manuscript.

For untrained animals, we dedicated littermates to assess the swimming behavior of pups at different time points (trials 1, 2, 3, and 4) during the first two postnatal days to compare with trained animals. We had to test a large number of animals to achieve a statistically comparable sample size.

3. In Fig. 1B1 and Fig. 1B2: the changes were strange: At trial 1 of untrained mice, the proportion of mice unable to swim at session 2 increased significantly compared with that at session 1. Does it mean that P1 pups lose swimming ability after a 45-second break?

We agree with the reviewer that this result is surprising, as the increased proportion of mice unable to swim during Trial 1, Session 2, is observed only in untrained pups but not in trained pups, even though it should be the same, given that it was also the trained pups' first exposure to water. A similar increase in the proportion of non-swimming animals between S1 and S2 is observed across the other three trials (2, 3, and 4), likely reflecting the impact of fatigue. We have no clear explanation for why the trained group did not exhibit the same decrease in S2 during Trial 1, except for the potential influence of interindividual variation and sample size.

4. The authors studied training-induced gene expression changes using RNA-seq at P3. The molecules involved in the key signaling pathways should be deeply analyzed combining with the electrophysiological characteristics and morphological changes of training-induced neurons. For example, downregulated DEGs were clustered in neurogenesis, synaptogenesis, developmental NMJ, et al. Which DEGs could support the findings in changes of electrophysiological and morphological characteristics after training? Likewise, upregulated DEGs were clustered in many important biological processes, and these results should be further illustrated and confirmed.

We fully agree with the reviewer that the description of the RNA-seq data in the initial version of our study lacked sufficient detail. In the revised manuscript, we now provide a comprehensive overview of the genes dysregulated following training. Supplementary Table 1 has been added to present all the DEGs found in the analysis. Figure 2 has been completely redesigned to display the various transcript families that show significant changes. These are illustrated through bar plots highlighting the different transcription factors, homeobox genes, synaptic and neurotransmission-related genes, as well as genes associated with apoptosis.

Corresponding updates have been made to the Results and Discussion sections to reflect these changes. The enrichment analyses for network processes, and Gene Ontology (GO) terms are now included as a supplemental figure (Fig. S1 in the revised manuscript). We have also removed panels A and B from Figure 4, as these results are now incorporated into the new Figure 2.

5. At page 21 Line 12-13: "The proportion of MNs expressing LTD, STD, or No Plasticity differed significantly between untrained and trained mice…" What conclusion could be made from this finding? The authors should summarize at the end of this paragraph.

The following sentence was added lines 515-517 in the result section describing the impact of training on synaptic plasticity: "This result suggests that training induced changes in the ADSP expressed at VLF-MN synapses, which are likely part of the functional adaptations triggered by this slight but significant increase in motor activity".

6. The authors described: a significant decrease in the amplitude of sEPSCs, less frequent but larger sIPSP events in trained MNs compared to the untrained MNs. In Fig. 6B2, the frequency of sIPSPs looked higher in the trained MNS (red dots) than the untrained MNs (black dots)? In Fig. 6B3, the amplitude of sIPSPs looked smaller in the trained MNS (red dots) than the untrained MNs (black dots)? Is there any morphological evidence supporting this finding?

The reviewer is completely right and the text has been corrected to correct this error in the Results section: "Moreover, we observed <u>more</u> frequent but <u>smaller</u> sIPSP events in trained MNs (n = 11) compared to the untrained MN cohort (n = 24; KS test, p = 0.0002 and 0.0008, respectively)."
We did not study whether these data are corroborated by morphological changes. As shown in the revised version of Figure 2, several DEGs correspond to transcripts coding for inhibitory neuron differentiation and development as well as for synaptic and scaffolding proteins. Changes in the expression of these proteins could therefore sustain the changes we observed in the network excitability. Histological and western blot analysis will be required to further address this question. The following sentence has been added in the Discussion lines 671-676: "Among the differentially expressed genes after training, we observed numerous transcripts coding for transcription factors involved in inhibitory neuron development and differentiation, as well as for synaptic and scaffolding proteins. Anatomical and biochemical analyses will be required to confirm changes in protein expression, but we hypothesize that these changes underlie at least part of the differences we observed in the spinal network excitability after training".

7. The authors found a notable increase of 5-HT and NA between P3 and P10 in trained mice, I was wondering if it was possible to present any differences of their fiber density using immunofluorescent staining spinal sections.

The HPLC analysis revealed an increase in 5-HT and NA content in the lumbar spinal cord of trained pups, but this increase may be entirely unrelated to fiber density, as it could result from enhanced monoaminergic synthesis or reduced degradation. Nonetheless, we fully acknowledge that histological quantification of monoaminergic fibers at P3 and P10, along with an analysis of serotonergic and NAergic receptors at the pre- and postsynaptic levels in lumbar motoneurons, would have been highly valuable. Our transcriptomic data identified differentially regulated genes encoding synaptic, scaffolding proteins as well as the alpha 1d adrenergic receptor. Investigating the effects of monoamines on motoneurons and the synaptic transmission they receive after training would provide further insight into the functional adaptations occurring in the developing spinal cord after training. Given the critical role of the serotonergic and noradrenergic systems as both neuromodulatory and trophic factors in the developing spinal cord, we believe this topic warrants a dedicated study beyond the scope of the present work.

The following sentences have been added in the discussion to address this point lines 788-792: "Our transcriptomic data identified differentially regulated gene encoding the alpha-1D adrenergic receptor. Given their essential role in the development of spinal motor networks, further investigations are required to understand the histological and functional adaptations that monoaminergic systems exhibit following motor training."

Reviewer #3: Review Plos Biology : PBIOLOGY-D-24-03358R1
Title: When Little Means a Lot: Impact of a Brief Early-life Motor Training on Mouse Neuromuscular Development.
Authors : Quilgars et al

Overall comments
The authors compared the impact of an early swim training (5 sessions of 15 sec, twice a day for 2 consecutive days) in neonatal mice pups, compared to untrained pups (2 sessions of 7 sec, twice a day for 2 consecutive days for swimming assessement) on several structural and functional features in the spinal cord and in the interactions between motneurons and muscles. Briefly, they found changes in the acquisition of swimming abilities (acceleration in trained pups), no changes in the intrinsic properties of motoneurons from the lumbar spinal cord except a few aspects of membrane excitability, changes in some features of currents and synaptic plasticity, down- and upregulated genes involved in membrane properties of motoneurons and others structural aspects, changes in some neurotransmitters, and alterations of axonal myelination in the motor regions of the spinal cord. They also found changes in the morphology, phenotype after such an early and short motor training. All the different features studied here are very interesting and correspond to a huge stack of data and work, but sound like a juxtaposition, so they need to be more linked together into an integrated whole study.
The study is very sound and interesting, well-conceived, well-done and well-written but several mistakes and typos are present. The Results part on swim motor training lacks of precision and details that need to be clarified (see description below), otherwise the remaining parts of the manuscript are sound. The manuscript needs more work, precision and clarifications since it really deserves to be published in Plos Biology with a high impact in the field of developmental neuroplasticity. One can deplore the absence of working hypothesis in the Introduction that may link and justify the methods used to test it, and the use of too many abbreviations. English and typos should be corrected.

The English and typos have been corrected using AI. We may be wrong, but in our opinion our working hypothesis seems to be materialized by the two questions we raised in the last paragraph of the Introduction: we use a combination of techniques to study, from genes to behavior, whether training induced an acceleration of maturation or an adaptation to increased motor activity in the neuromuscular system. It is true that our study can be seen as a catalog of techniques and that each result at each level of the neuromuscular system will require further studies to precisely investigate the impact of training, but our aim in this work was to determine whether all the different factors have been impacted.

Abstract
From the Results and Fig 1A, the faster acquisition of swimming was assessed at P1 and P2 in trained and untrained pups; please clarify timing of results.

Please see the response below regarding the swim motor training

Methods

The description of the statistics used and their choice should be increased and justified. For example, I don't understand the use of Chi square in many comparisons and make some statistical results unclear, like the numbers of motoneurons expressing STD, LTD or no plasticity. Statistical test description needs to be added in the Results, like the value of Mann-Whitney tests and Spearman…

The following sentences have been added lines 311-315 at the end of the material and methods section: "Mann-Whitney or T-test, depending on the normal distribution of the data, were used to compare two datasets. Chi-square was used for contingency analysis, Kolmogorov-Smirnov for spontaneous synaptic events, and two-way ANOVA followed by uncorrected Fisher's LSD for further comparisons. All data are expressed as means ± SEM. Statistical significance level was set at $p < 0.05$."

All the statistical tests used have been indicated in the results section, in the legends of Figures and in Tables 3-4 and 5.

Results

Part 1: "Motor training accelerated the acquisition of the four-limb motor pattern for swimming."

The Methods, Results, Fig 1 from A to D and the corresponding legend are unclear and hard to follow and decipher, so they need to be clarified as this part is crucial for understanding the whole manuscript.

Based on the comments of the reviewer, we think that there is a misunderstanding in the training protocol we used.

Two groups of mice were compared to assess the impact of the motor training we have developed and were used for all the different experiments presented in this study (behavioral analysis, transcriptomic, electrophysiology and histology):

- trained mice submitted to 5 sessions of 15 s with 45 s pause each day at 9 am and 5 pm at both P1 and P2

- untrained mice were tested only once either at P1 at 9 am, or at P1 at 5pm or at P2 at 9 am or at P2 at 5pm. This is why the color of the different mice presented in the "untrained mice" panel in Figure 1A are different and why we indicate mouse 1, mouse 2…etc. Each trial was made with different mice.

The sentence lines 111-112 in the Methods Section was changed to clarify that point: " Untrained mice were tested only once at one time point corresponding to one of the training time slots (P1 9 am or P1 5pm or P2 9 am or P2 5pm), with each test consisting of two 7-second sessions separated by a 45-second interval (Fig. 1A)" and in the legend of Figure 1 line 1055: "Untrained mice were tested only once either at P1 9 am or P1 5pm or P2 9 am or P2 5pm during two swimming sessions of 7 seconds each (S1 green box and S2 red box)".

In Cazalets et al (1990), is written page 127: "During the first 24 hours, only 2/3 of the [rat] pups were swimming, whereas at day 2 they were all able to swim and move around the tank. … As all of them [rat pups] were not able to swim at P0, we did not perform quantitative analysis." The patterns of swimming of mice pups seems critical to be better defined in the present study, compared to rats that served as reference in previous reports (Cazalets et al, 1990) and from the "patterns H0 to H2" in mice pups in Cazalets (2000). Do the patterns of swimming differ between the various strains of mice used in the present study and from "C3H control strain" in Cazalets et al (2000). This is preponderant and crucial for the rest of the study. In addition, the authors use two strains of mice in the present study, the use of each one in the different experiments needs also to be clarified.
How many mice were used in the present study? Why don't the authors show also the numbers of mice for scores 2 to 4? Why the numbers in session 1 but not in session 2? All this part on the "motor training" and "swimming patterns" needs to be clarified from the Methods, Results and corresponding Fig 1 and legend.

We have removed the reference to Cazalets (1990 and 2000) when describing the motor score we used, as it differs significantly from the one used in those studies. In their work, the motor score was based solely on hindlimb use, whereas we chose a motor score that considers all four limbs and the rostrocaudal gradient of limb use. As stated in the Results section, using C57BL/6J mice, we observed that as early as P1, the majority of mouse pups were already capable of swimming with their two forelimbs and even with one or both hindlimbs. These findings contrast sharply with the results of the two studies of Cazalets and collaborators, in which rats were almost unable to swim at P1—a difference that aligns with the greater immaturity of rats compared to mice at birth. C3H mice appear to exhibit some differences in swim pattern development. They are fatter than C57BL/6J mice with smaller litter sizes. Given the already dense nature of our manuscript, we have chosen not to discuss this point, despite its relevance, as highlighted by the reviewer.

The numbers above the bars in Figures 1B1 and 1B2 now represent the total number of animals tested in each trial (for untrained mice) or training session (for trained mice) and are consistent across different sessions (S1 and S2 for untrained animals, and S1 to S4 for trained animals). We fully agree that this was not clear in the first version of our manuscript, as the numbers for trained animals were sometimes positioned above the bars of the 0 or 1 score and did not include all the animals tested. We apologize for this mistake. This has been corrected in the revised version of Figure 1.

As stated in the manuscript (lines 332), we considered only well-defined swimming patterns for analysis, excluding animals that exhibited mixed patterns during the 7-second motor scoring period. This explains why the number of animals included in the analysis varies across training sessions.

For all experiments in this study, we used C57BL/6JRJ mice, except for the transcriptomic analysis. HB9-GFP C57BL/6JRJ mice (n = 10) were used to identify the lateral motor column

during laser microdissection but were not included in the behavioral analysis, although we did assess whether training affected their swimming behavior.

Why not to add videos of patterns in supplemental data? Supplemental videos may clarify the "non well-defined patterns of swimming" and each pattern of swimming.

We completely agree that providing videos of the different swimming patterns would have been highly beneficial. Unfortunately, we did not film the pups during all sessions and are therefore unable to provide recordings of all observed swimming patterns. Since completing this experimental series, our lab has relocated to a new building with a new animal facility, and our research focus has shifted to amyotrophic lateral sclerosis on the $SOD1^{G93R}$ mouse line. Consequently, we have not resumed breeding C57BL6/J mice. To provide additional videos, we would need to purchase two littermates solely for filming, which we hope the reviewer will agree is not ethically justifiable. The only available recordings were made at the end of the training sessions. In the revised version of our manuscript, we have included one video providing the direct comparison between the swimming pattern of a P3 trained pup and a P3 untrained animal (supplemental video 1).

What is the interest of fig Fig 1C?

Figure 1B allows for a direct comparison of the evolution of the swimming pattern between trained and untrained mouse pups. Figure 1C illustrates the progression of the swimming pattern in trained mice across the five different sessions of the four training periods. This figure shows that, starting from session 4, a fatigue effect is observed during training 3, which justified our decision to limit the training to five sessions (see also our responses to Reviewer 2 regarding the training protocol setup).

The numbers of mice used appears for the body weight comparison. It should appear before in the manuscript, as well as the repartition of the two strains, as previously suggested.

The sentence has been revised line 94 to : Experiments were conducted using 538 C57Bl/6JRJ from Janvier (Le Genest-Saint-Isle, France) and 10 B6.Cg-Tg(Hlxb9-GFP)1Tmj/J (HB9-GFP) mice from the Jackson laboratory (Bar Harbor, ME, USA), aged postnatal day 1 (P1) to P12, without sex discrimination.

The maximal duration of the righting reflex session should also be described in Methods for ethical consideration since the authors wait for rats to be exhausted in this task (i.e. time to abandon in Fig1D).

The sentence lines 130-131 has been revised to “The trial was considered "abandoned" if the animal failed to turn, ceased moving, and did not exceed 90 seconds.”

The conclusion of this part of the Results may include the difference of training between untrained that performed 7 sec of swimming twice, 2 times a day for 2 consecutive days.

Please see the responses above.

Actually, the part on top of Fig 1A for untrained mice (Trial 1 - Mouse 1 and so on...) is not clear and does not correspond to any description in the legend, Methods or Results. It suggests that each trial was made with different mice. This needs to be clarified.
The difference in swim training between the two groups needs to be clarified and emphasized along the manuscript. I don't understand why the authors used untrained control rats to assess swimming at P1 and P2 (corresponding to a "training") while they did it later from P5 to P14, which may have been enough and leaving untrained control pups really untrained?

Please see the responses above. A large number of pups were sacrificed at P3 for the transcriptomic, electrophysiological and histological approaches used in this paper. Four littermates were dedicated to the study of “the long lasting effects” of the training in P5-P14 mice. These littermates were submitted to the same protocol ie trained mice submitted to 5 sessions of 15 s with 45 s pause each day at 9 am and 5 pm at both P1 and P2 and untrained mice were tested only once either at P1 at 9 am, or at P1 at 5pm or at P2 at 9 am or at P2 at 5pm.

Table 1 on Motor Scores. Why is there no score of 1, as it could be 0 for no swim and 1 for swim with 1 limb? Please justify and clarify.
The authors describe the exclusive use of hind limbs around P15, so why do the use of the hind and forelimbs are mixed in scoring from P5 to P12? Actually, the gradation of scores of this scale is not completely clear; please justify.

The motor score of 1 is absent from our results because, between P1 and P2, mice never swim using only one of their four limbs. As previously mentioned in our responses, the motor scores we used take into account all four limbs rather than only the hind limbs, as was done previously by Cazalets and collaborators.

We adapted the motor score based on our observations of the swimming behavior of C57BL/6 mice: at P1, they could swim using only their two forelimbs, their two forelimbs plus one hind limb, or directly with all four limbs. Between P5 and P12, pups use all four limbs to swim and progressively transition to a swimming pattern using exclusively their two hind limbs, with an intermediate period during which one forelimb may be used along with the two hind limbs.

In our opinion, the two motor scores we used are more detailed and discriminative than the one previously used for rat and C3H mouse pups. Table 1 was modified to clarify that point and the following sentence lines 114““To evaluate swimming ability, we visually scored the number of limbs used during the first 7 seconds of each session (see Table 1 P1-P3).” has been revised to “To evaluate swimming ability, we visually scored the number and types of limbs used during the first 7 seconds of each session (see Table 1 P1-P3).”

Part 2: "Motor training induces changes in gene expression in the lateral motor column."

There are discrepancies in the numbers of genes up -and downregulated between text and Fig 2.

The correction has been done.

The description of the changes in genes is too short and lacks of details to get the insights, only a summary of the results is produced. Maybe more details could be provided in Supplemental data? This raises the question of its interest as it has not been justified previously.

We fully agree with the reviewer that the description of the RNA-seq data in the initial version of our study lacked sufficient detail. This point was also raised by reviewer 1. In the revised version of our manuscript, we now provide a comprehensive overview of the genes dysregulated following training. Supplementary Table 1 has been added to present all the differentially expressed genes (DEGs) found in the analysis. Figure 2 has been completely redesigned to display the various transcript families that show significant changes. These are illustrated through bar plots highlighting the different transcription factors, homeobox genes, synaptic and neurotransmission-related genes, as well as genes associated with apoptosis. Corresponding updates have been made to the Results and Discussion sections to reflect these changes. The enrichment analyses for network processes, and Gene Ontology (GO) terms are now included as a supplemental figure (Fig. S1 in the revised manuscript). We have also removed panels A and B from Figure 4, as these results are now incorporated into the new Figure 2.

To me, there are also discrepancies between statistics in the text corresponding to the Fig 4C2 and C3. Please clarify.

We have reviewed this section, and it appears to us that the statistical analysis reported in the text (two-way ANOVA) corresponds to Figures 4C2 and 4C3. The inward component of the current was not significantly different between trained and untrained motoneurons. However, the outward component of the current, both under control conditions and in the presence of Ba^{2+} in the bath, was significantly different between trained and untrained motoneurons. In both trained and untrained motoneurons, the inward and outward components of the current were found to be significantly different, as is typically observed for the IH current.

Last part of Results: "Early motor training impacts the development of postural control during the two first postnatal weeks"
I don' agree with authors on the summary of results: "These data indicate that the postural development of trained pups differs slightly but significantly from that of untrained ones during the first postnatal week and that these differences disappear during the second postnatal week." To me, these results appear stronger than they described and should be emphasized more positively as an improvement and acceleration of the acquisition of sensorimotor abilities, as written in the Abstract.

Discussion

The different parts are very interesting and well-written and suited, but to me, several positive results are underestimated, like the "absence" of changes in the muscle phenotype and others. As previously suggested, all these very interesting results lack of integration and links together to serve a hypothesis on the impact of such an early and short motor training on so many structural, morphological and functional properties for the development and exercise-driven plasticity of the sensorimotor system.

Although the training protocol we used was brief, it undoubtedly had an impact, but not all the parameters we examined were significantly altered. Furthermore, the effects of the training disappeared during the second postnatal week. For this reason, we were very cautious not to overestimate or oversell our results. As previously mentioned, we fully acknowledge that our discussion and more generally the whole manuscript may seem like a catalog. We could have tried to link the changes observed in motoneuron membrane properties, synaptic plasticity, and neuromuscular junction morphology, for example, with the behavioral changes we observed. The primary aim of this study was to demonstrate that even minimal manipulation of the neuromuscular system could impact its development and alter it, and it is in this context that we shaped our discussion.

These comments are meant to help the authors to improve the quality of the manuscript, which deserves for me to be published in Plos Biology
